# Association of marital status with DNA methylation-based biological age among US older adults

**DOI:** 10.3389/fpubh.2025.1535524

**Published:** 2025-06-10

**Authors:** Xian Cui, Jianghua Huo, Yaqian Xu, Hui Zhang, Chongyu Ding, Jun Du, Xiangwei Li

**Affiliations:** ^1^Diagnostic Imaging Center, Shanghai Children's Medical Center, School of Medicine, Shanghai Jiao Tong University, Shanghai, China; ^2^School of Public Health and Management, Jiangsu Medical College, Yancheng, Jiangsu, China; ^3^School of Global Health, Chinese Centre for Tropical Diseases Research, Shanghai Jiao Tong University School of Medicine, Shanghai, China

**Keywords:** marital status, DNA methylation, epigenetic clock, biological age acceleration, public health

## Abstract

**Background:**

DNA methylation (DNAm) algorithms, such as GrimAge and GrimAge2, have emerged as reliable measures of biological age acceleration and may offer additional insights into health outcomes. While previous research has explored the relationship between marital status and mortality, its association with accelerated biological aging remains understudied. This study aimed to examine the relationship between marital status and DNAm-based biological age acceleration in older adults.

**Methods:**

Data from the National Health and Nutrition Examination Survey (NHANES) were used to assess GrimAge and GrimAge2 in 2,420 U.S. adults aged 50 and older. DNAm profiles were measured using the Infinium Methylation EPIC BeadChip kit (Illumina). Linear regression models, adjusted for potential confounders, were used to estimate associations between marital status and DNAm-based biological age acceleration.

**Results:**

Significant associations were observed between marital status and biological age acceleration. After adjusting for potential confounders, never-married males had significantly higher GrimAge2Acc (*β* = 0.95, 95% CI: 0.17–1.73) and GrimAgeAcc (*β* = 0.88, 95% CI: 0.28–1.47) scores compared to married males. Additionally, widowed females exhibited significantly higher GrimAge2Acc (*β* = 0.43, 95% CI: 0.02–0.85) compared to their married counterparts.

**Interpretation:**

This study highlights the significant role of marital status in biological aging, particularly for men. Never-married status may be linked to higher risks of biological aging, highlighting the need for social and environmental interventions that promote healthier lifestyles and emotional support for older adults, especially those without partners. These findings underscore the importance of addressing social determinants of health to mitigate the adverse effects of marital status on biological aging and overall health outcomes.

## Introduction

In recent decades, substantial evidence has demonstrated an association between marital status and health, with unmarried individuals experiencing higher mortality risks (1–3) and an increased likelihood of developing cancer (4–6). This association may be partially explained by the fact that being in a committed relationship is globally linked to healthier lifestyle behaviors, including reduced smoking and alcohol consumption, improved diet, increased physical activity, and better weight management (7, 8), all of which are known to influence aging (9). Additionally, some studies have reported that living singly, regardless of the reason, is associated with shorter leukocyte telomere length (LTL), a biomarker of biological aging (10, 11).

Biological age, which reflects an individual’s physiological age and overall health status, can differ from chronological age. It accounts for lifestyle, environmental influences, and genetic predispositions, providing a more comprehensive understanding of the aging process and its health impacts (12). Epigenetics, which regulates gene expression without altering the DNA sequence, links genetic factors and environmental influences, making it crucial for studying biological aging (13). There is a lack of consensus on definitions and measures of biological aging rates, posing challenges in aging and health research (14). Epigenetic clocks, such as GrimAge (15) and GrimAge2 (16), offer a promising solution by providing accurate estimates of biological age based on DNA methylation (DNAm) patterns (17). These clocks strongly predict lifespan and healthspan, offering a more nuanced understanding than traditional biomarkers (17–19). These two metrics, also known as second-generation epigenetic age clocks, were demonstrated superiority in predicting health outcomes and mortality compared to other tools like PhenoAge (17–19). GrimAge incorporates a wide range of aging-related factors, including methylation data from 1,030 CpGs, plasma proteins, and smoking pack-years (7), making it a comprehensive measure of biological age. The residuals from the regression of GrimAge and GrimAge2 on chronological age, termed GrimAgeAcc and GrimAge2Acc, have been shown to be strongly associated with age-related clinical outcomes and mortality (17, 19).

Limited observational evidence has suggested that certain socioeconomic status, lifestyle behaviors may be related to GrimAgeAcc and GrimAge2Acc, including marital status (20, 21). Given the established link between marital status and healthier lifestyle behaviors, it is hypothesized that marital status may be associated with these epigenetic aging measures. However, research investigating the extent of this association remains limited.

In this study, we aimed to investigate the associations between marital status and GrimAgeAcc and GrimAge2Acc in 2,420 U.S. adults aged 50 and older, using data from the National Health and Nutrition Examination Survey (NHANES).

## Methods

### Study population and data collection

This cross-sectional study utilized data from the NHANES, conducted by the National Center for Health Statistics in two-year cycles beginning in 1999, to assess the health and nutritional status of the U.S. population (22). For the current analysis, data from the 1999–2000 and 2001–2002 cycles were employed. Each cycle operates independently, recruiting distinct cohorts, with study protocols approved by the institutional review board of the National Center for Health Statistics and the Centers for Disease Control and Prevention (CDC). Written informed consent was obtained from all participants. Given that this analysis used de-identified data without direct participant interaction, it was classified as exempt from institutional review board review in accordance with National Institutes of Health policy. The completed STROBE (Strengthening the Reporting of Observational Studies in Epidemiology) checklist for cross-sectional studies is provided as Supplementary Table 1.

As previously described (23, 24), demographic information (chronological age, sex, education) was collected through self-report questionnaires. Race and ethnicity were recorded using predefined categories to characterize the population and ensure adequate representation of non-Hispanic Black and Mexican American individuals. Height and weight measurements were taken, and health behaviors, including alcohol consumption and smoking, were documented. Smoking status was classified as never, former, or current smoker. Alcohol consumption was categorized as yes/no based on the question: “In any one year, have you had at least 12 drinks of any type of alcoholic beverage?” One drink is defined as 12 oz. of beer, 4 oz. of wine, or 1 oz. of liquor.

**Figure 1:**
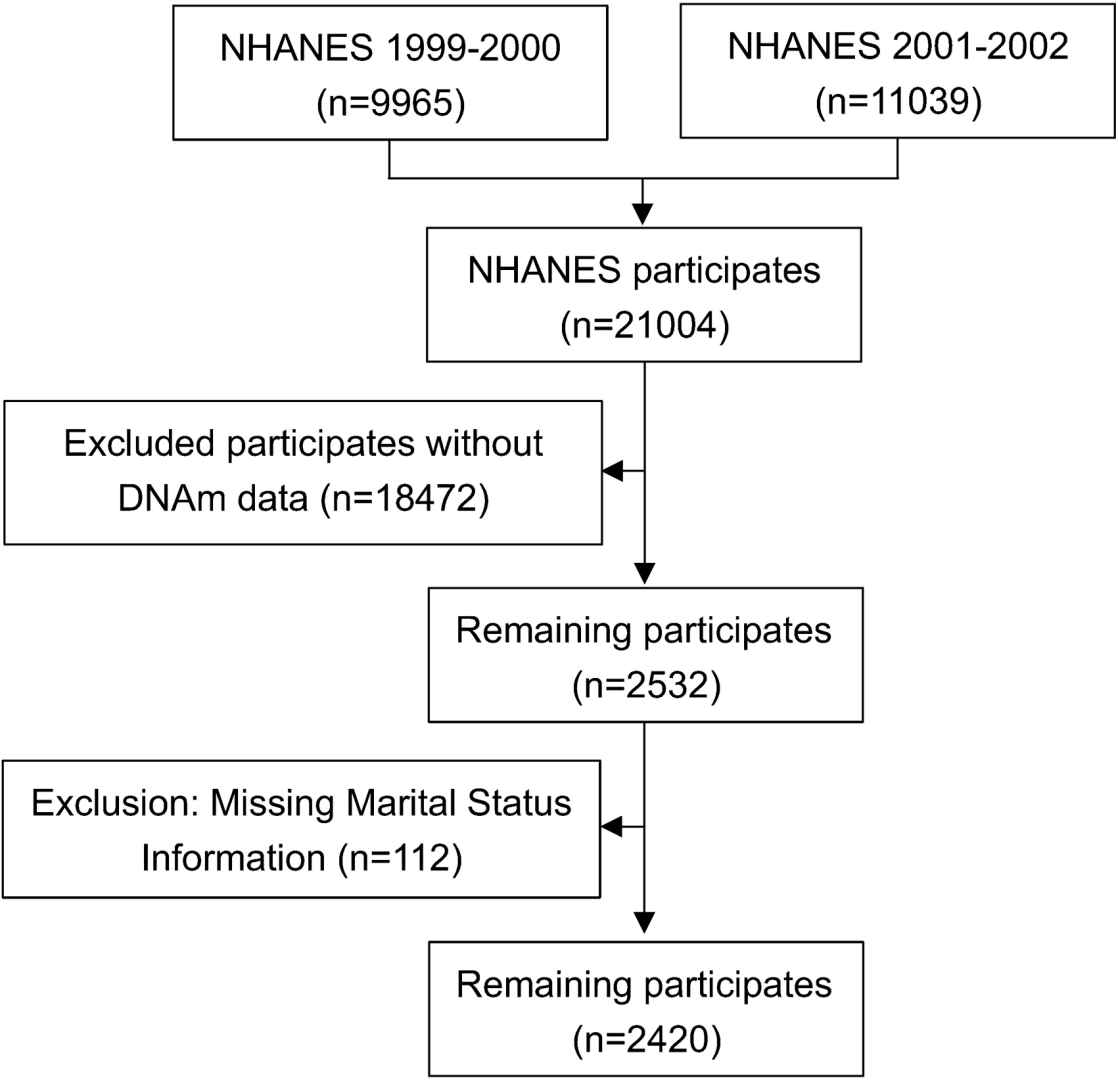
Flowchart of the participant selection process. NHANES, National Health and Nutrition Examination Survey.

The analysis utilized data from the 1999–2000 and 2001–2002 National Health and Nutrition Examination Survey (NHANES) cycles. From an initial pool of 21,001 participants across both cycles, 18,472 individuals were excluded because they lacked DNA methylation (DNAm) data, which was only accessible for adults aged 50 years or older. A further 112 participants were excluded due to missing marital status information. After applying these exclusions, the final analytical sample comprised 2,420 individuals. A detailed flowchart of the participant selection process is provided in Figure 1.

### Marital status ascertainment

Marital status was collected through self-report questionnaires and classified into six categories: widowed, divorced, separated, never married, married, and living with a partner.

To ensure that our analysis accurately reflects the broader population of U.S. adults aged 50 and older, we carefully considered the representativeness of our sample. The NHANES is designed to be nationally representative, and we utilized the survey weights provided by NHANES in our analyses. These weights account for the complex survey design and ensure that our estimates are representative of the U.S. population. Specifically, we applied these weights in our linear regression models to adjust for potential biases arising from the overrepresentation or underrepresentation of certain marital status groups. This approach helps to mitigate the impact of sample stratification on our results and enhances the generalizability of our findings.

### DNA methylation assessment and calculation of DNAm algorithms

As outlined in the NHANES DNA Methylation Array and Epigenetic Biomarkers Data Documentation (25), DNAm measurements were performed on a subset of adults aged 50 years and older who participated in the 1999–2000 or 2001–2002 NHANES cycles and had blood samples collected for DNA purification. This subset included approximately half of the eligible non-Hispanic White participants, along with all eligible non-Hispanic Black, Mexican American, other Hispanic, and participants of other racial backgrounds.

DNAm was assessed using the Infinium Methylation EPIC BeadChip kit (EPIC, Illumina, San Diego, CA, United States), following the manufacturer’s instructions, in Dr. Yongmei Liu’s laboratory at Duke University. In brief, the raw fluorescence intensity data from BeadChip arrays were processed in the RStudio environment using the minfi, lumi, and wateRmelon R packages. The data underwent background subtraction and color correction to account for technical biases, with control probes on the BeadChip being utilized. Samples were excluded as outliers if their median intensity values for both methylated and unmethylated channels were below 10.5.

The DNAm-derived biomarkers GrimAge (15) and GrimAge2 (16) analyzed in this study were obtained as precomputed values from the NHANES database. These established epigenetic aging measures were originally developed through multi-stage analytical processes. GrimAge was constructed by first deriving DNA methylation surrogates for seven plasma proteins and smoking history, then combining these predictors with chronological age using mortality-calibrated coefficients (15). The enhanced GrimAge2 algorithm expanded the predictive model to incorporate 1,163 CpG sites (compared to GrimAge’s 1,030 sites) with refined biomarker estimates and updated mortality risk weights (16). For our analyses, we further computed age-adjusted residuals (GrimAgeAcc and GrimAge2Acc) by regressing each epigenetic measure against chronological age to isolate mortality-associated variance independent of chronological aging.

### Statistical analysis

All analyses incorporated adjustments for the complex survey design of NHANES, including sampling weights (calculated per NHANES protocols), stratification variables, and clustering effects unless explicitly stated otherwise. Demographic characteristics of the study subjects at baseline were summarized by subset using standard descriptive methods. We then compared GrimAgeAcc and GrimAge2Acc values across different marital status groups to examine potential differences between these categories.

Next, linear regression models were employed to evaluate the associations between marital status and both GrimAgeAcc and GrimAge2Acc, adjusting for age, sex, leukocyte composition (estimated by the Houseman approach (26)), body mass index (BMI, kg/m^2^), smoking pack years (estimated using a DNAm-based proxy, the Maas 13-CpGs model (27)), alcohol consumption (never, ever, and current), annual family income, and survey years.

Additionally, subgroup analyses stratified by sex were performed, and the statistical significance of interactions with sex was evaluated by incorporating interaction terms into the models, with GrimAgeAcc and GrimAge2Acc treated as continuous variables.

All analyses were performed using SAS, version 9.4 (SAS Institute, Inc., Cary, NC). Statistical significance was defined by *p*-values <0.05 in two-sided testing.

### Role of funding source

The funders did not have any role in study design, data collection, data analyses, interpretation, writing of report, or decision to publish the study.

## Results

### Sociodemographic characteristics

Table 1 summarizes the sociodemographic characteristics of the 2,420 participants. Males accounted for 51.15% of the sample, with a mean age of 66.01 ± 9.88 years, similar to the mean age of females at 66.42 ± 10.22 years. Approximately 40% of both males and females identified as Non-Hispanic White, and nearly 45% had less than a high school education. Around half of the participants reported an annual income of $24,999 or less.

**Table 1:** Characteristics of study population by sex.

Characteristics	Males (*N* = 1,238)	Females (*N* = 1,182)	Total (*N* = 2,420)
Age (years; mean ± SD)	66.01 ± 9.88	66.42 ± 10.22	66.20 ± 10.05
Race/ethnicity			
Non-Hispanic white	513 (41.44)	480 (40.61)	993 (41.03)
Non-Hispanic black	259 (20.92)	259 (21.91)	518 (21.40)
Mexican American	352 (28.43)	324 (27.41)	676 (27.93)
Other Race – Including Multi-Racial	36 (2.91)	39 (3.30)	75 (3.10)
Other Hispanic	78 (6.30)	80 (6.77)	158 (6.53)
Body mass index (kg/m^2^, mean ± SD)	28.15 ± 4.78	29.14 ± 6.37	28.63 ± 5.63
Educational levels (*N*/%) ^a^			
Less than high school	554 (44.75)	549 (46.45)	1,103 (45.58)
High school diploma	222 (17.93)	274 (23.18)	496 (20.50)
More than high school	461 (37.24)	357 (30.20)	818 (33.80)
Smoking pack-years (*N*/%) ^b^	22.60 ± 13.67	15.32 ± 11.38	19.04 ± 13.11
Alcohol consumption			
Current	80 (6.46)	323 (27.33)	403 (16.65)
Ever	147 (11.87)	302 (25.55)	449 (18.55)
Never	959 (77.46)	489 (41.37)	1,448 (59.83)
Unknown	52 (4.20)	68 (5.75)	120 (4.96)
Physical activity^c^			
Inactive	147 (11.87)	91 (7.70)	238 (9.83)
Active	1,023 (82.63)	978 (82.74)	2001 (82.69)
Unable to do activity	68 (5.49)	112 (9.48)	180 (7.44)
Annual family income			
$ 0 to $ 24,999	585 (47.25)	512 (43.32)	1,097 (45.33)
$25,000 to $74,999	439 (35.46)	447 (37.82)	886 (36.61)
$75,000 and over	109 (8.80)	124 (10.49)	233 (9.63)
Unknown	105 (8.48)	99 (8.38)	204 (8.43)

a Data missing for 3 participants.

b DNA methylation predicted pack years of smoking.

^c^Vigorous or moderate activity, data missing for 1 participant.

### Marital status and DNAm algorithm means

Table 2 shows DNAm algorithm means by gender and marital status, with distributions visualized in Supplementary Figure 1. Among the 1,238 males, approximately three-quarters were married, while around 8% were widowed or divorced. Only 22 males (2.67%) reported never being married. Among females, 48.05% were married, 29.36% were widowed, 12.27% were divorced, and 1.69% were never married.

**Table 2:** Means of DNAm biological age acceleration by gender and marital status.

Marital status	*N* (%)	Age (years; means ± SD)	GrimAge2Acc (means ± SD)	GrimAgeAcc (means ± SD)
Male	1,238	66.01 ± 9.88	7.41 ± 6.03	1.92 ± 5.39
Married	926 (74.80)	66.00 ± 9.70	6.96 ± 5.84	1.51 ± 5.20
Widowed	103 (8.32)	74.70 ± 8.33	6.10 ± 5.67	0.88 ± 5.16
Divorced	99 (8.00)	62.90 ± 8.14	10.37 ± 6.52	4.55 ± 6.08
Separated	28 (2.26)	61.75 ± 8.85	11.13 ± 6.39	5.41 ± 5.73
Living with partner	49 (3.96)	60.31 ± 8.78	9.13 ± 5.90	3.33 ± 4.99
Never married	22 (2.67)	60.48 ± 8.92	9.34 ± 6.66	3.84 ± 5.76
Female	1,182	66.42 ± 10.22	4.59 ± 5.63	−1.56 ± 4.96
Married	568 (48.05)	63.84 ± 9.12	4.78 ± 5.39	−1.34 ± 4.69
Widowed	347 (29.36)	73.50 ± 9.36	2.90 ± 5.26	−3.17 ± 4.64
Divorced	145 (12.27)	63.19 ± 8.90	6.23 ± 6.23	0.12 ± 5.46
Separated	42 (3.55)	61.69 ± 7.82	5.94 ± 5.26	−0.83 ± 4.72
Living with partner	60 (5.08)	62.75 ± 9.70	6.46 ± 6.15	0.18 ± 5.47
Never married	20 (1.69)	60.95 ± 8.21	8.13 ± 5.20	1.14 ± 5.08

DNAm, DNA methylation; GrimAge2Acc, DNA methylation GrimAge2 acceleration; GrimAgeAcc, DNA methylation GrimAge acceleration; SD, standard deviation.

### Pairwise comparisons of means of DNAm algorithms by marital status

**Figure 2:**
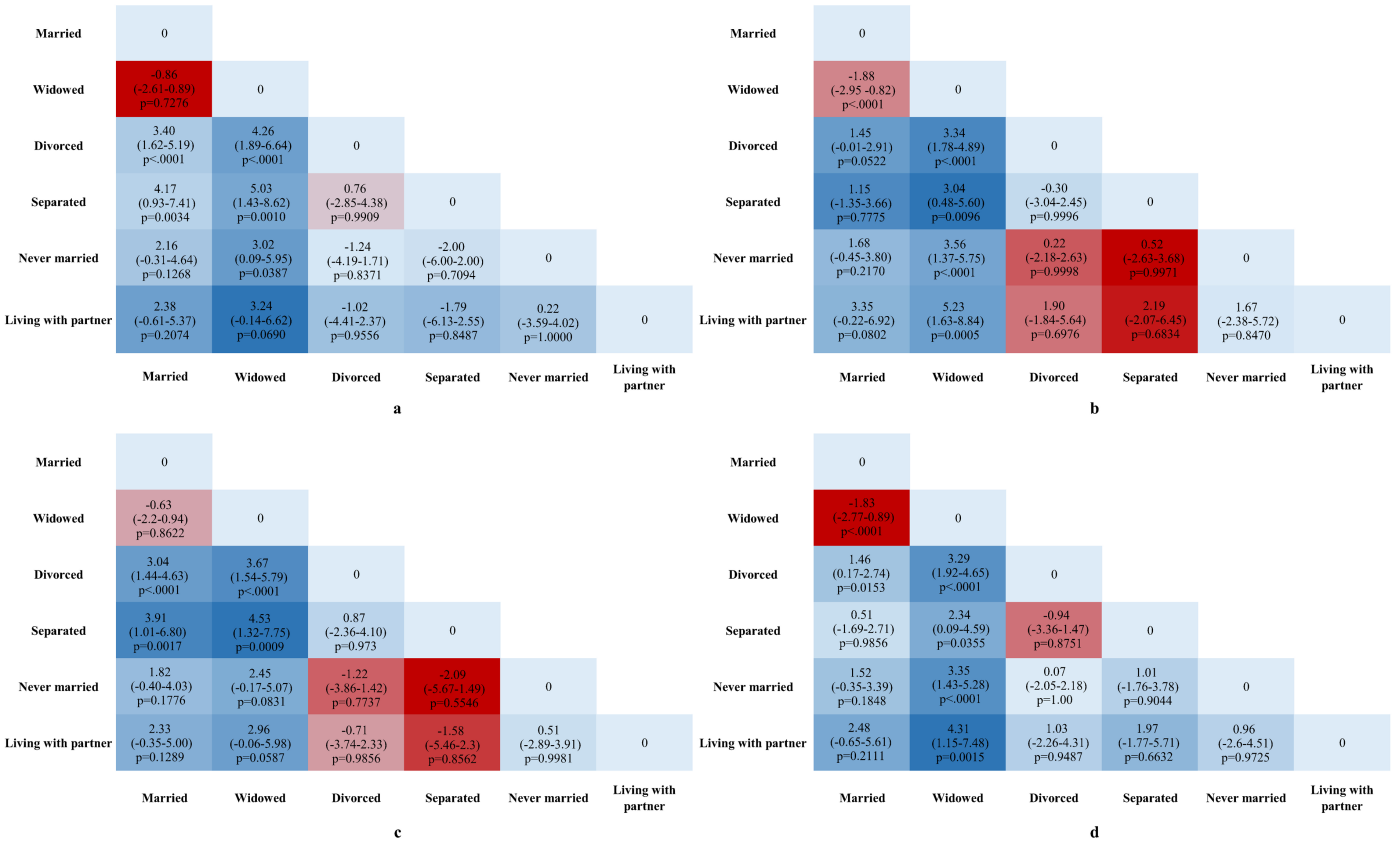
Mean Difference in GrimAgeAcc and Grim2AgeAcc across various marital statuses. All values represent pairwise group mean differences with 95% CIs in GrimAgeAcc and GrimAge2Acc years. **(a)** Pairwise comparisons of GrimAge2Acc across various marital statuses in males. **(b)** Pairwise comparisons of GrimAge2Acc across various marital statuses in females. **(c)** Pairwise comparisons of GrimAgeAcc across various marital statuses in males. **(d)** Pairwise comparisons of GrimAgeAcc across various marital statuses in females. Effect sizes (*β*) and 95% confidence intervals (CI) are annotated directly on the plots. *p*-values are indicated for significant comparisons (*p* < 0.05).

Figure 2 shows pairwise comparisons of mean difference of DNAm algorithms by sex and marital status. Among males, divorced and separated individuals exhibited significantly higher GrimAge2Acc and GrimAgeAcc scores compared to their married counterparts (*p* < 0.01). In contrast, widowed females had significantly lower GrimAge2Acc and GrimAgeAcc scores in most comparisons, except when compared to separated females regarding GrimAgeAcc, where no significant difference was found (*p* < 0.05).

### Multivariable analysis of marital status and DNAm algorithms

**Figure 3:**
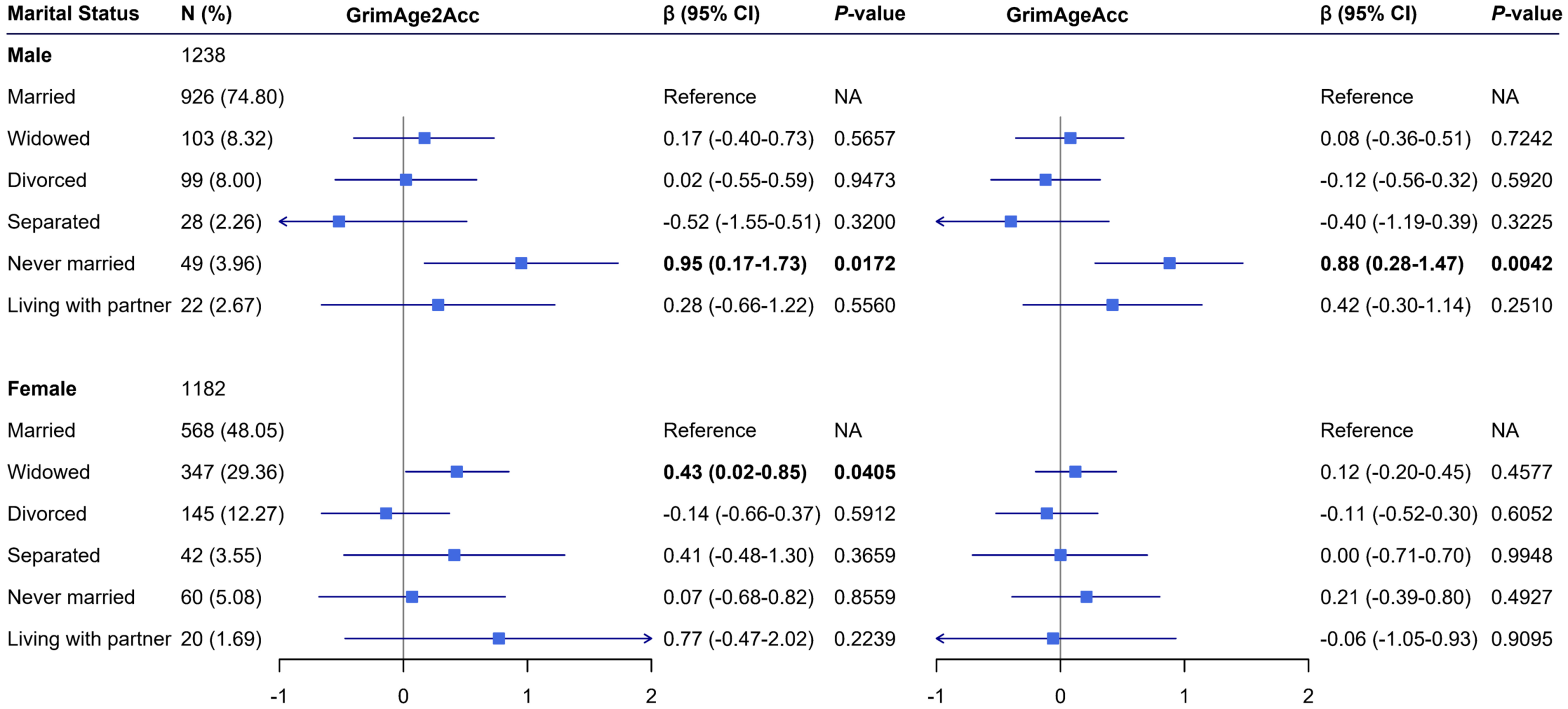
Associations of varying marital status with DNAm biological age accelerations. Adjusted *β* coefficients and 95% CI for each marital status category compared to married individuals. *p*-values are indicated for significant associations (*p* < 0.05). CI, confidence interval; GrimAge2Acc, DNA methylation GrimAge2 acceleration; GrimAgeAcc, DNA methylation GrimAge acceleration; NA, not available. Models were adjusted for age, race/ethnicity, leukocyte composition, body mass index, educational level, smoking pack-years, alcohol consumption, physical activity, annual family income, and survey years.

Figure 3 displays the associations between marital status and DNAm algorithms, stratified by sex. After adjusting for potential confounders, never-married males had significantly higher GrimAge2Acc (*β* = 0.95, 95% CI: 0.17–1.73) and GrimAgeAcc (*β* = 0.88, 95% CI: 0.28–1.47) compared to married males. Among females, widowed women had significantly higher GrimAge2Acc (*β* = 0.43, 95% CI: 0.02–0.85) compared to married women.

## Discussion

In this study, we investigated the association between marital status and two epigenetic aging measures-GrimAgeAcc and GrimAge2Acc-among 2,420 U.S. adults aged 50 and older using data from the NHANES. Our findings indicate that marital status is significantly associated with epigenetic aging biomarkers, and the impact of marital status on these biomarkers varies by sex.

Our study found that never-married males exhibited significantly higher GrimAgeAcc and GrimAge2Acc scores compared to their married counterparts, suggesting that a lack of marital status might be linked to accelerated epigenetic aging in males. These findings align with prior research indicating that unmarried individuals tend to experience poorer health outcomes and shorter life expectancy, potentially due to lifestyle factors such as higher rates of smoking, alcohol consumption, and physical inactivity, as well as social isolation and limited social support networks (9, 10, 28). The higher GrimAgeAcc and GrimAge2Acc observed among never-married males may reflect these lifestyle-related factors that are known to influence biological aging processes (29, 30). In line with these results, Wang et al. (31) found that unmarried individuals exhibited greater acceleration in other DNAm aging algorithms, such as PhenoAge acceleration (32) and DunedinPACE (33), compared to those married or cohabiting. In our study, we extended these findings by using DNA methylation-based aging measures, which are more advanced predictors of biological age, and by including a larger sample of older adults with a broader range of marital status categories, providing a more nuanced understanding of how marital status associated with aging over time.

For females, the findings were more nuanced. Widowed women had significantly higher GrimAge2Acc scores compared to married women, suggesting that the loss of a spouse-a major life stressor-may accelerate epigenetic aging. This is consistent with research suggesting that widowhood, particularly in older age, is associated with increased emotional and physical stress, which could contribute to biological aging through mechanisms such as inflammatory responses and oxidative stress (34, 35). Interestingly, the association between marital status and GrimAge was less pronounced among divorced and separated females, which could imply that the psychological and physiological associated with of marital status vary depending on the context and duration of the relationship (36, 37).

The relationship between marital status and epigenetic aging may be mediated by various factors, including lifestyle behaviors, psychological well-being, and social support. Marriage and long-term partnerships often foster healthier behaviors, such as reduced smoking, better dietary habits, and increased physical activity (38, 39). In contrast, individuals who live alone, especially those who are widowed or never married, may experience higher levels of stress, social isolation, and loneliness, which are known to negatively affect health and accelerate aging (40). Additionally, social support from a spouse or partner has been shown to buffer the effects of stress, potentially mitigating the biological processes that contribute to aging (15, 37). For never-married males, role theory (41) suggests that marriage traditionally provides men with clearly defined social roles and responsibilities that promote healthier behaviors and regular social engagement. Without these structural benefits, unmarried men may experience greater biological vulnerability due to weaker social integration and poorer health self-regulation. This aligns with our findings of significantly accelerated epigenetic aging in never-married males compared to their married counterparts.

The differences observed between males and females also suggest that gender-specific factors may play a role in how marital status associated with epigenetic aging. For example, males may benefit more from the protective effects of marriage in terms of lifestyle factors, while females might be more vulnerable to the negative effects of widowhood due to emotional and psychological stressors related to loss and changes in social support networks (36). This supports the idea that marriage may confer protective benefits by facilitating healthier lifestyles, reducing harmful habits, and promoting emotional stability.

The stress-buffering hypothesis (42) helps explain why widowed females show more pronounced epigenetic aging effects than males. Women often serve as primary emotional caregivers in marriages, so widowhood may represent not just the loss of a spouse but also the collapse of their central social role and support network. This dual loss may trigger stronger biological stress responses, potentially explaining their elevated GrimAge2Acc scores. Conversely, divorced/separated women show less epigenetic aging than widows, possibly because divorce represents an active life transition rather than an irreplaceable loss (43). These patterns may also reflect broader gender norms in health behaviors (43). Married men typically benefit from wives’ health-promoting influences (e.g., meal preparation, medical appointment reminders), while women often maintain health behaviors regardless of marital status. This could explain why unmarried men show greater biological aging consequences than unmarried women. Future research should examine how changing gender roles in modern relationships may modify these associations.

Although the study suggests a promising association between marital status and lifestyle behaviors, the field still lacks more definitive evidence to establish this relationship in a causal manner. It is important to acknowledge in the manuscript that many of the studies exploring the association between marital status and lifestyle behaviors are observational. Moreover, the interconnection between eating habits and marital status is indeed multifaceted. Changes in life situation, such as divorce or widowhood, are often accompanied by emotional stress that can lead to changes in eating habits, such as increased consumption of processed foods or decreased dietary variety. Therefore, it is important to address and discuss that eating behavior can, in many cases, be an adaptive response to the emotional and social circumstances faced by an individual. Future research should investigate how specific diets, eating patterns, and the nutritional quality of meals relate to marital status and how this, in turn, may influence biological aging, given its strong relationship with epigenetics.

A major strength of this study is its use of a large, nationally representative cohort, which enhances the generalizability of the findings. Additionally, the use of advanced epigenetic aging biomarkers, such as GrimAgeAcc and GrimAge2Acc, provides a more accurate measure of biological age than traditional markers, offering a more nuanced understanding of how marital status associated with aging. However, several limitations must be noted. First, while we adjusted for important confounders, such as lifestyle factors (e.g., smoking and alcohol use), residual confounding may persist due to unmeasured psychosocial factors such as depression, social support networks, or caregiving stress. Additionally, the absence of detailed information on preexisting diseases and health conditions is a recognized limitation. These factors can significantly impact biological aging, and their exclusion may lead to biased results. Future studies should consider incorporating detailed health histories and comprehensive psychosocial assessments to provide a more comprehensive understanding of how marital status and lifestyle habits interact to influence health and aging. Second, the cross-sectional nature of this study limits the ability to establish causal relationships between marital status and epigenetic aging. Longitudinal studies are needed to further explore how changes in marital status over time may impact biological aging. Third, we cannot rule out potential reverse causation, whereby accelerated biological aging may influence marital dynamics rather than vice versa. These limitations highlight the need for future longitudinal research incorporating comprehensive psychosocial assessments to better understand the complex interplay between marital factors and epigenetic aging processes. Fourth, while the NHANES is designed to be nationally representative, our sample included a higher proportion of married males and widowed females. This stratification may limit the generalizability of our findings to other populations with different marital status distributions. To address this, we utilized NHANES survey weights in our analyses to adjust for potential biases arising from the overrepresentation or underrepresentation of certain marital status groups. However, the use of survey weights cannot fully eliminate the potential for sampling bias.

Our findings suggest that marital status is associated with epigenetic aging, with distinct effects observed between males and females. These results underscore the importance of considering marital status as a potential factor influencing biological aging and highlight the potential benefits of interventions aimed at reducing social isolation and promoting healthier lifestyle behaviors. Future research should explore the mechanisms underlying these associations and investigate whether interventions targeting marital status or social support could positively impact epigenetic aging. Additionally, studies examining the impact of changes in marital status over time will be critical for understanding the long-term effects of relationship transitions on biological aging. This study also emphasizes the potential protective benefits of maintaining marriage, which may confer advantages through the sharing of economic, behavioral, and psychosocial resources, ultimately contributing to healthier lifestyles and reduced harmful habits, particularly in men.

## Data Availability

Publicly available datasets were analyzed in this study. This data can be found here: https://www.cdc.gov/nchs/nhanes/about_nhanes.htm.
